# Explorations in Reported Moral Behaviors, Values, and Moral Emotions in Four Countries

**DOI:** 10.3389/fpsyg.2021.661172

**Published:** 2021-04-30

**Authors:** Liisa Myyry, Klaus Helkama, Mia Silfver-Kuhalampi, Kristina Petkova, Joaquim Pires Valentim, Kadi Liik

**Affiliations:** ^1^Faculty of Educational Sciences, University of Helsinki, Helsinki, Finland; ^2^Faculty of Social Sciences, University of Helsinki, Helsinki, Finland; ^3^Institute of Philosophy and Sociology, Bulgarian Academy of Sciences, Sofia, Bulgaria; ^4^Faculty of Psychology and Educational Sciences, University of Coimbra, Coimbra, Portugal; ^5^School of Natural Sciences and Health, Tallinn University, Tallinn, Estonia

**Keywords:** guilt, moral behavior, secrets, shame, values

## Abstract

University students (*n* = 758) from Bulgaria, Estonia, Finland, and Portugal were given a list of morally relevant behaviors (MRB), the Schwartz Value Survey (PVQ40) and Tangney’s TOSCA, measuring empathic guilt, guilt over norm-breaking, and shame. A factor analysis of MRB yielded 4 dimensions: prosocial behaviors, interpersonal transgressions, antisocial behaviors and secret transgressions. Prosocial behaviors were predicted by self-transcendence–self-enhancement (SET) value contrast only while the three transgression categories were associated with both SET and openness to change–conservation (hedonism–conformity) contrast. Norm-breaking guilt was more strongly associated with behaviors than were empathic guilt and shame. However, shame was (positively) associated with secret transgressions in three countries, after controlling for values. The associations were strongest in Bulgaria and Estonia while fewer associations were found in Finland and Portugal. The implications of the findings for the cross-cultural psychology of morality are discussed.

## Introduction

The relation of values to behavior has most usually been addressed by assessing behaviors that are conceptually, *a priori*, related to the corresponding values (e.g., following religious ceremonies–tradition; helping victims of distant disasters–universalism; e.g., Bardi and Schwartz, 2003; Lönnqvist et al., 2006; Schwartz and Butenko, 2014; Schwartz et al., 2017). Schwartz et al. (2017) suggested a need for studies that focus on behaviors that are of particular interest in themselves. This study follows that suggestion by focusing on morally relevant behaviors (MRB). We ask how and to what extent values are associated with *a posteriori* formed categories of MRB.

We generated a list of behaviors and asked the respondents in four countries to report whether they had engaged in them. Self-reports of behavior are not necessarily accurate (Fischer, 2017). As values are part of our identity and we want to appear consistent, values and reported behaviors are more closely related than values and real or other-reported behaviors (e.g., Bardi and Schwartz, 2003; Schwartz, 2016). The human tendency to act in a socially desirable manner and the common-method biases also influence the value-behavior relations (Podsakoff et al., 2003). In this paper, our primary concern is not the truthfulness of the reports. The primary question is: if we find that (morally relevant) behaviors *x*, *y*, and *z* occur together in the reports of Bulgarian, Estonian, Finnish, and Portuguese respondents, what are the factors that link these behaviors together? Do the categories reflect dimensions of value priorities? To what extent are they related to different moral emotional tendencies, e.g., to feel guilt or shame? How do values and moral emotional tendencies relate to one another? And how are values and moral emotional tendencies associated with another in explaining MRB–do guilt or shame proneness add explanatory value after values are taken into account? Finally, are there cross-national differences in the strength of the value-behavior and moral emotions-behavior associations?

Schwartz’s (1992) theory of universal content and structure of values defines values as motivational constructs, cognitive representations of abstract goals, which serve to define situations, elicit more specific goals, and guide action. Values are organized into 10 universal types (more recently divided into sub-types Schwartz et al., 2012) that serve different interests or motivational goals. Values, their contents, and (exemplary) items are as follows:

*Power*: societal prestige and controlling others (social power, authority, and wealth).

*Achievement*: personal success and competence according to social standards (successful, capable, and ambitious).

*Hedonism*: pleasure and satisfaction of sensual needs (pleasure, enjoying life).

*Stimulation*: excitement, novelty and challenge in life (daring, varied life, and exciting life).

*Self-direction*: independent action and thought, making one’s own choices (freedom, creativity, and curious).

*Universalism*: understanding, tolerance and protection for the welfare of all people and for nature (broadminded, social justice, equality, and protecting environment).

*Benevolence*: protecting the welfare of close others in everyday interaction (helpful, honest, forgiving, and responsible).

*Tradition*: respect, commitment, and acceptance of the customs and ideas that one’s culture or religion imposes on the individual (humble, devout, and accepting my portion in life).

*Conformity*: restraint of actions, inclinations and impulses likely to upset or harm others, or violate social expectations or norms (polite, obedient, honoring parent and elders).

*Security*: safety, harmony, and stability of society, of relationships and of self (national security, family security, social order, and clean).

In Schwartz’s model, the goals and interests that values serve can be either compatible or conflicting with each other. The values form a two-dimensional continuum, organized along a circular structure consisting of two main dimensions, self-transcendence vs. self-enhancement and openness to change vs. conservation. Self-transcendence refers to motivation to transcend selfish concerns and promote the welfare of others (benevolence and universalism values). Self-enhancement comprises values, which motivate people to further their own personal interests even at the expense of others (power and achievement values). Openness to change values refer to motivation to follow one’s own intellectual and emotional interests (self-direction, stimulation, and hedonism), whereas conservation values refer to preferring the *status quo* and the certainty provided by relationships with close others, institutions and traditions (tradition, conformity, and security values). The circular continuum reflects motivational compatibility and conflict among values so that the more compatible any two values are, the closer they are on the circle. Values located on the opposite sides of the circle are in conflict. This motivational continuity is manifested in the sinusoid form of the magnitudes of correlations both among the values and between the values and other variables as one moves along the circle.

From the point of view of morality, values can be categorized into two broad categories: conservation values inhibit antisocial behavior or justify resistance to temptation to deviate from moral norms and self-transcendence values promote or justify prosocial, altruistic actions (Helkama, 2004, 2011; Vauclair et al., 2014; Miles and Vaisey, 2015). For instance, in a large representative sample of United States citizens, Miles and Vaisey (2015) found three morally relevant factors: (1) order included three Schwartz values: conformity, tradition, and security, (2) other-focus included Schwartz’s benevolence and universalism values, and (3) self-focus consisted of the five Schwartz openness to change and self-enhancement values (self-direction, stimulation, hedonism, achievement, and power). On the other hand, each of the 10 or 19 Schwartz values could be considered as so many virtues of their own (Schwartz et al., 2017).

Studies of value-behavior relations (see Jiga-Boy et al., 2016; Schwartz, 2016; Roccas and Sagiv, 2017 for reviews) have typically relied on two paradigms. In one the focus is on the relation of values to corresponding behaviors, e.g., benevolence and helping, or tradition and following traditions. Initiated by Bardi and Schwartz (2003), studies in this paradigm give participants a list of behaviors that are conceptually related to the Schwartz values, and ask them and/or their partners or peers to report how often they have engaged in those behaviors. In the experimental version of this paradigm, participants’ performance in a task designed to measure behavior corresponding to a value [e.g., prosocial behavior in a game and universalism value; Lönnqvist et al. (2013)] is related to their value priorities. Another variant of this approach is to focus on behaviors that instantiate a given value and, for example, on cross-cultural variation in the instantiations of given values (see Maio, 2016; Hanel et al., 2018).

It has been a common assumption that when normative pressures are strong, values do not predict behavior or attitudes. Normative pressure could be due to value importance or behavior frequency (Schwartz, 2016) so that higher correlations have been found for relatively unimportant values and for infrequent behaviors. A case in point are gender roles. Myyry and Helkama (2001) examined the relations of values to empathy and found that the links were much stronger among males than females for whom empathy is part of the gender role. Value-behavior relations are also moderated by individuals’ orientation to norms. Those who regard conformity values as important are less likely to behave according to their (other) values. This has been found in several studies, first by Lönnqvist et al. (2006) then replicated by Koivula (2008) and Lönnqvist et al. (2009). However, Schwartz et al. (2017) failed to find support for the hypothesis that normative pressure weakens value–behavior relations, and the question under what conditions this effect occurs remains open.

The second paradigm starts from a certain behavior and examines the values that predict it. For instance, political activism or engaging in risky sexual behavior (multiple partners, no condom) are best predicted by universalism and hedonism, respectively. Typically, the pattern of value-behavior correlations follows the sinusoid form implied by the notion of motivational continuity. Political activism is positively predicted by values that are adjacent to universalism, viz., self-direction and stimulation, and negatively by values that are opposite to universalism. Given the motivational continuity of the Schwartz Value Model, the highest positive and highest negative correlations of a certain behavioral or attitudinal variable are not necessarily with the exact opposite values, e.g., universalism and power, respectively, but might also be with an adjacent value, e.g., universalism and achievement. However, most of the patterns for the 30 value–behavior/attitude relations reviewed by Schwartz (2016) followed a sinusoidal or quasi-sinusoidal pattern. Recently, Bilsky et al. (2020) found that attitude toward norms among both delinquents and non-delinquents was best predicted by the conformity–hedonism contrast. There are some exceptions to the sinusoid pattern of motivational homogeneity of behavior, though.

Goodwin et al. (2002) found that risky sexual behavior was best positively predicted by hedonism, stimulation and the non-adjacent power values (and negatively by security and non-adjacent universalism and benevolence). Thus, risky sexual behavior was motivationally heterogeneous, a mixture of pleasure-seeking vs. own safety and domination of others vs. concern for their welfare and dignity. The pattern for law abiding has been equally heterogenous, with two bipolar contrasts, power vs. universalism/benevolence and hedonism vs. conformity (Benish-Weisman et al., 2017).

While most of the value-behavior patterns reported in the literature have been bipolar, i.e., to the highest positive correlation of a value with a behavior corresponds the highest negative correlation of the opposite value, a unipolar pattern was found for artistic occupations by Knafo and Sagiv (2004) in a study of occupations. Artistic occupation correlated negatively with conformity values but did not show a positive correlation with any value. Thus, those working in the artistic domain tend to reject conformity values but may have a variety of values to which they are committed.

As mentioned at the outset, Schwartz et al. (2017) pointed out the need for the second type of studies, which focus on theoretically interesting behaviors. Schwartz et al. (2017) raise one further issue that we address here. They found that the pattern of value-behavior correlations was largely bipolar. Schwartz calls them tradeoffs between values that promote behavior and values that inhibit it. One of the intriguing findings was that the values expected to propel behavior correlated more strongly and consistently with behavior than did the values expected to inhibit it. We may ask whether the stronger association of promoting vs. preventing values with behavior is a general characteristic of value-behavior associations. Our design where behavior categories are inductively derived is apt to answer this question.

In the study of guilt and shame, there is a lot of confusion and controversy (see, e.g., the reviews by Silfver-Kuhalampi et al., 2013 and Dempsey, 2017). In this study, we used Tangney’s approach and her measure (Tangney and Dearing, 2002), in which guilt is seen as negative evaluation of one’s actions and reparative behavior as its criterion is stressed. In contrast, shame is defined as a negative evaluation of a global self, with a tendency to hide or escape. Some researchers have pointed out that the TOSCA measures specific, somewhat narrow forms of guilt and shame (Luyten et al., 2002; Giner-Sorolla et al., 2011), which is important to consider when interpreting the results. When the TOSCA is examined with factor analysis, the highest loading items on the guilt scale refer to reparative behavior, and therefore is has been questioned to what extent this scale measures emotion instead of behavioral tendencies. On the other hand, prosocial motivation is empirically so strongly associated with other elements of guilt that this distinction does not really seem to exist (Silfver-Kuhalampi et al., 2013, 2015). In terms of shame, the TOSCA shame scale emphasizes aspects of negative self-esteem, feeling worthless and bad as a person. Some studies have pointed out that the meaning of shame differs between languages and cultures: in some languages the translation-equivalent term for shame refers to embarrassment-like experiences, in others the meaning of shame is closer to guilt (Wallbott and Scherer, 1995; Kollareth et al., 2018). Therefore, it is important to remember that the TOSCA measures mainly a form of shame that focuses on self-image and a sense of self-esteem and to a lesser extent to the public and social aspects of shame (a distinction that has been pointed out by many researchers, for example Gausel and Leach, 2011). Shame measured by the TOSCA has been found to relate to poor self-regulation (Woien et al., 2003) and depression (Dempsey, 2017).

Most items in the TOSCA involve consequences for other people, but some items tap guilt and shame over norm violations that have no direct consequences for others. The patterns of correlations of empathy and guilt with values are similar for universalism and benevolence but differ for conformity, which does correlate with guilt but shows very low or non-existent correlations with empathy (Silfver et al., 2008). Helkama et al. (2018) tested the hypothesis that this discrepancy is due to the “pure” norm violation items and modified the TOSCA by separating the consequences-for-others items as a measure of empathic guilt, and by adding a few pure norm violation items. They found that the new measure of empathic guilt did not correlate with conformity whereas the norm-breaking guilt did so–and showed its highest correlation with conformity. Moreover, the opposite values, hedonism and stimulation were negatively correlated with it, and the pattern of correlations followed the sinusoidal form. The modified TOSCA was used in the present study.

Shame as measured by the TOSCA has shown far less systematic associations with values than has guilt, but positive correlations with tradition and conformity and negative correlations with self-direction and power have been found (Silfver et al., 2008; Helkama et al., 2018). Guilt is associated with prosocial behavior (Tignor and Colvin, 2019).

It seems plausible to assume that people are more likely to behave according to their values in individualistic and egalitarian societies than in collectivistic, embedded and hierarchical ones (Schwartz, 2008; Hofstede et al., 2010). Embedded cultures emphasize in group solidarity, social order, respect for tradition, and security. In hierarchical ones, important values are social power, authority, humility, and wealth. In spite of similar labels, the Hofstede and Schwartz dimensions are not very closely related (power distance and hierarchy *r* = 0.41, collectivism and embeddedness *r* = 0.64; Smith et al., 2006, p. 46). Table 1 shows the scores of the four target countries on the relevant Hofstede and Schwartz dimensions. Finland scores low on Hofstede’s power distance and high on individualism. Bulgaria was chosen to represent a European country which is the opposite on those two dimensions, high on power distance and low on individualism. The contrast is similar on Schwartz’s hierarchy and embeddedness. The third country, Portugal, is interesting because on Hofstede’s (Hofstede et al., 2010) dimensions it seems to be similar to Bulgaria as a high power distance and low individualism country, but according to the corresponding Schwartz dimensions it looks similar to Finland, as it is low on both hierarchy and on embeddedness. With the exception of Estonia, the Hofstede scores are based on measurements carried out in the late 60s, while the Schwartz’s scores derive from the early 90s. We suspect that the rapid socio-economic development in Portugal since its regime shift in 1974 and membership in the EEC in 1986, with the accompanying value changes might explain the large discrepancy between the Hofstede and Schwartz scores for Portugal. Thus, based on Schwartz dimensions we expect that patterns of relationships would be similar in Finland and Portugal, and Bulgaria would show a different pattern. From Hofstede’s scores the prediction would be that the connections are stronger in the individualistic Finland, and weaker in high power distance and collectivistic Bulgaria and Portugal. Estonian scores were estimated in the early 2000s, and they suggest that Estonia and Finland are quite close to one another on power distance and individualism, whereas on the Schwartz dimensions (defined by measurements from the 1990s) Estonia and Bulgaria appear to be quite similar.

**TABLE 1:** Bulgaria, Estonia, Finland, and Portugal compared on cultural value dimensions.

Dimension	Bulgaria	Estonia	Finland	Portugal
Power distance (Hofstede)	70	40	33	63
Hierarchy (Schwartz)	2.68	2.04	1.80	1.89
Individualism (Hofstede)	30	60	63	27
Embeddedness (Schwartz)	3.87	3.81	3.37	3.43
Tightness–looseness (Uz)	60.4	55.4	74.5	87.4

*Ranges: power distance: 104–11; hierarchy: 3.49–1.49; individualism: 91–6; embeddedness: 4.63–3.11; tightness-looseness: 3.4–126.*

Another possible explanation for cultural variation could be cultural tightness or looseness. This dimension, initially suggested by Pelto (1968), and more recently developed by Gelfand et al. (2011) and Uz (2015), refers to the normative pressures in a culture. Tight cultures have many strong norms and low tolerance of deviant behavior, whereas in loose cultures social norms are weak and tolerance of deviant behavior is high. Gelfand et al.’s (2011) measure is based on the perception of citizens of the strictness of norms and intolerance of deviations in their country. Gelfand et al. (2011) do not report scores for Bulgaria or Finland on this dimension, but Portugal is a tight culture (score 7.8) and Estonia a loose one (2.6). Uz’s (2015) approach is based on the variation of values, norms and behaviors (measured by their standard deviation) in a country. Table 1 indicates that on Uz’s index, Estonia is the tightest and Portugal the loosest of the four countries. Thus, the Gelfand et al. (2011) measure and the Uz measure of cultural tightness are not consistent with regard to our target countries.

In their review of cross-cultural variability of value-attitude linkages, Boer and Fischer (2013) put forward a further viewpoint: the strength of the linkages may depend not only on the properties (individualism, power distance etc.) of a culture but also on the nature of the value–psychological variable linkage. Thus, they expected and found that the correlations of the self-transcendence–self-enhancement value dimension with variables associated with care and fairness would be higher in individualistic cultures (in this case Estonia and Finland, possibly Portugal) than in collectivistic ones (in this case Bulgaria, possibly Portugal). Boer and Fischer also found support for the hypothesis that collectivism is related to stronger conservation–relevant variables links.

Of course, it is possible to define some of those culture-level variables (individualism, tightness) from the samples, which do not necessarily reflect the national average differences.

To summarize our research questions, we wanted, first, to explore the nature of the value-behavior relations from the viewpoint of moral values, by looking at behaviors that in the reports and minds of our respondents group together. Moral values are exemplified by their two functions, promotion of other people’s welfare (Self-Transcendence) and inhibition of doing bad things, in the sense of following norms (Conservation values, most clearly conformity). To what extent do we find correlational patterns that follow the main axes, Self-Enhancement–Self-Transcendence and Conservation-Openness to Change, as has usually been the case in previous studies, to what extent patterns in which the main motivational contrasts are mixed, as in risky sexual behaviors (Goodwin et al., 2002)?

Second, we examine the question, raised by Schwartz et al. (2017), whether the values that (conceptually) promote behavior are more strongly associated with behavior than are values inhibiting it.

The third focus are the associations of the moral behavior categories with tendencies to feel empathic or norm-breaking guilt and shame and the role of values and moral emotions in explaining the reported frequencies of different categories of MRB. More specifically, we examine the question whether taking emotional variables into account adds any explanatory power beyond values.

A fourth focus is cultural variation in the strength of value-behavior-emotion links. While the four target countries, Bulgaria, Estonia, Finland, and Portugal are all members of the EU, they have differing historical backgrounds and recent histories, which are reflected in the contradictory scores on such cross-cultural value dimensions as individualism or cultural tightness–looseness. Our data provide an opportunity to compare the variation in the light of cross-cultural typologies.

The research procedure followed the principles for research with human participants and the study did not involve elements requiring ethical review (Finnish Advisory Board on Research Integrity 2019). The respondents provided an oral informed consent to participate the study and they were informed that they could withdraw their participation any time without any reason.

## Materials and Methods

The sample^1^ consisted of 758 university students in the fields of psychology and social sciences. They were recruited from Sofia (Bulgaria, *n* = 166), Tallinn (Estonia, *n* = 239), Helsinki (Finland, *n* = 151), and Coimbra (Portugal, *n* = 202). The percentage of female respondents was 82% (67% in Sofia, 82% in Tallinn, 89% in Helsinki, and 89% in Coimbra), and the samples differed in terms of gender (χ^2^(3) = 36.80, *p* < 0.001). The mean age for the whole sample was 23.5 years (sd = 5.7). Mean ages for the subsamples were: Sofia 21.3 (sd = 1.5), Tallinn 27 (sd = 6.9), Helsinki 25 (sd = 5.8), and Coimbra 20.0 (sd = 2.3). Because the variances of age were not homogeneous, we conducted a non-parametric Kruskal–Wallis test to examine whether age differed between the samples. The test revealed that age varied between the countries (χ^2^(3) = 389.24, *p* < 0.001). The multiple comparisons using a Mann–Whitney Test with Bonferroni adjustment showed that Finnish respondents were older than respondents in Bulgaria and Portugal (both *p*_s_ < 0.001) and younger than in Estonia (*p* < 0.05). The age of Bulgarian respondents differed significantly from the age of Portuguese and Estonian respondents (both *p*_s_ < 0.001). Thus, further analyses are controlled for gender and age.

The respondents filled out in class a questionnaire consisting of demographic questions, the Schwartz et al. (2001) Portrait Values Questionnaire (PVQ-40), and a modified Tangney and Dearing (2002) TOSCA (adults) measure of guilt and shame, and the checklist of MRB, in that order.

### Measures

#### Morally Relevant Behaviors

A panel of Finnish graduate and post-graduate students and post-doctoral researchers in psychology and social psychology generated, in a “brainstorming” session, a list of MRB. The instruction was to forget moral psychological theories and just try to find a set of everyday behaviors that were meaningful and more or less likely to occur in college students’ life. The definition of each item took into account the likelihood of each behavior so that for some behaviors (e.g., cheating on exam) the wording was “ever” whereas for behaviors that were supposed to occur more often, it was “within the past year” or “6 months” or “2 months.” The response alternatives were “not possible” (0), “no” (1), “yes” (2). The list of behaviors was translated from English/Finnish into Bulgarian, Estonian, and Portuguese. A principal component analysis with Varimax rotation yielded seven factors with eigenvalues greater than 1 accounted for 52.51% of the total variance. Based on the Scree plot we ended up to limit the number of factors to four with a cutoff 0.40 for inclusion of a variable in a factor. The four factors, accounted for 35.14% of the variance. In all, as many as 13 of the original 30 items were discarded, either because of the high frequency of “not possible” responses or because they failed to load on the factors. The final list of behaviors is shown in Table 2 (Since it is not easy to buy fair trade bananas in Portugal, MB 20 was replaced in Portugal by “Over the last 2 months, did you buy anything in a Fair Trade store”).

**TABLE 2:** Morally Relevant Behaviors scale.

MB01.	Do you ever jump a queue?
MB02.	Have you ever broken bottles in nature?
MB03.	Have you ever cheated on an exam?
MB04.	Have you ever moved a thing lying on a road out of the way?
MB05.	Have you lent money (more than 20 EUR) to a friend within the past 6 months?
MB06.	Have you spoken ill of a friend within the past 6 months?
MB08.	Have you broken a promise without a good reason within the past 6 months?
MB12.	Have you ever done voluntary work?
MB13.	Have you ever poked around in secret among your friend’s or partner’s things?
MB14.	Have you ever lied to get sex?
MB15.	Do you sort out organic waste?
MB17.	Have you ever deceived your partner?
MB19.	Have you helped to lift a pram to a bus within the past 6 months?
MB20.	Have you bought fair trade bananas within the past 2 months?
MB21.	Have you ever read someone’s diary without permission?
MB23.	Have you ever urinated in a passageway?
MB27.	Have you ever read your friend’s or partner’s text messages in secret?
MB28.	Have you intervened on behalf of a person being bullied within past 2 years?
MB29.	Have you gone with two persons simultaneously without their knowing of each other? (i.e., courtship)
MB30.	Have you hit somebody within the past 2 years?

*Next a few questions about your concrete behaviors. Circle the appropriate alternative. If the behavior has not been possible for you, choose 0 = not possible, 1 = no, 2 = yes.*

#### Value Priorities

The Portrait Value Questionnaire (PVQ-40) (Schwartz et al., 2001) was used to measure value priorities. It contains items describing persons with different value priorities, e.g., “Success is important for him/her. (S)he wants to impress other people” (achievement). The respondent indicates how similar (s)he is with the person The PVQ consists of 40 items, describing different persons in terms of their goals, aspirations and wishes that point implicitly to the importance of a value. For instance, “Thinking up new ideas and being creative is important to her. She likes to do things in her own way” describes a person for whom self-direction values are important. For each item, participants respond to the question: “How much like you is this person?”, using a six-point scale (1 = not like me at all to 6 = very much like me). Each value is measured by 4–6 items. The alpha reliabilities for the values averaged 0.72, 0.62, 0.69, and 0.67 for Bulgaria, Estonia, Finland and Portugal, respectively.

#### Guilt and Shame

The version of Tangney’s TOSCA (adults) (Tangney and Dearing, 2002) we used consisted of 10 scenarios, e.g., “You make a big mistake on an important project at work. People were depending on you and your boss criticizes you.” Respondents rate the likelihood of reacting with four responses: shame (“I want to hide”) and guilt (“I should have done a better job”), as well as with externalization or detachment, using a five-point scale (1 = not probable 5 = very probable). In this study, empathic guilt scores were based on responses to six scenarios involving negative outcomes for others. The alphas for empathic guilt: Bulgaria 0.60, Estonia 0.47, Finland 0.58, and Portugal 0.56. Norm-breaking guilt was defined on the basis of four scenarios (breaking an object, in the original TOSCA, plus 3 additional ones, not paying TV license, taking a free ride in the underground, and walking against red light). Norm-breaking guilt alphas were 0.60 in Bulgaria, 0.47 in Estonia, 0.51 in Finland, and 0.21 in Portugal. Shame was calculated across all items. Alphas were satisfactory (0.61–0.70) except in Portugal (0.39). In spite of the low reliabilities in the Portuguese sample, we used the norm guilt and shame measures in the analyses, because all the items of these subscales correlated positively with the total score and made sense conceptually.

All measures for which there was no previous translation were translated from English into Finnish, Bulgarian and Portuguese and back translated to English by native bilinguals.

## Results

The means and standard deviations of the main variables are shown in Table 3. The table indicates, first, that in terms of the hierarchy of values, the Bulgarian sample differed from the other samples (rho = 0.58–66), which were similar to one another (rho = 0.89–0.93). Second, in terms of the tightness-looseness, calculated as the mean standard deviation of the ten values, the Portuguese sample was the tightest (Msd = 0.181), followed by Finland (0.196) and Estonia (0.197). In the loosest, Bulgarian, sample, the mean sd was 0.222. For the norm-breaking guilt, the tightest was again Portugal and the loosest Bulgaria, with Finland and Estonia in the middle. Because the variances of values were not homogenous across samples, a non-parametric Kruskal–Wallis test was conducted to examine differences in values between samples. The tests revealed that all value scores except self-direction and security differed according to the country: universalism χ^2^(3) = 111.072, *p* < 0.001; stimulation χ^2^ (3) = 33.723, *p* < 0.001; hedonism χ^2^ (3) 27.805, *p* < 0.001, achievement χ^2^(3) = 72.936, *p* < 0.001; power χ^2^(3) = 77.088, *p* < 0.001; conformity χ^2^ (3) = 27.747, *p* < 0.001; tradition χ^2^ (3) = 48.968, *p* < 0.001; and benevolence χ^2^(3) = 59.052, *p* < 0.001. The multiple comparisons using a Mann–Whitney Test with Bonferroni adjustment showed that compared to Estonian sample, Bulgarian students had lower scores in universalism, conformity and benevolence and higher scores in hedonism and achievement (all *p*_s_ < 0.001). Comparing to Finnish sample, Bulgarians had higher scores in stimulation, achievement and power and lower scores in universalism and benevolence (all *p*_s_ < 0.001). They also showed higher scores in stimulation, achievement and power and lower scores in universalism, tradition and benevolence than Portuguese students (all *p*_s_ < 0.001). Estonian and Finnish samples differed in stimulation, power, conformity (higher in Estonia, *p*_s_ < 0.001) as well as in universalism and benevolence (higher in Finland, *p*_s_ < 0.001). Estonians also showed higher scores in stimulation, power, conformity and lower scores in hedonism and tradition than Portugese sample (all *p*_s_ < 0.001). Samples from Finland and Portugal differed in universalism, benevolence and tradition, the former two being higher in Finland and tradition in Portugal (*p*_s_ < 0.001).

**TABLE 3:** Mean importance, standard deviation and rank of values and moral emotion variables in four countries.

Value	Bulgaria		Estonia		Finland		Portugal	
Universalism	1.03^a,b,c^ (0.16)	6.	1.13^a^ (0.16)	3.	1.24^b^ (0.19)	1.	1.13^c^ (0.14)	3.
Self-direction	1.19 (0.20)	1.	1.18 (0.16)	1.	1.20 (0.18)	3.	1.16 (0.16)	2.
Stimulation	1.06^a,b^ (0.30)	5.	1.08 (0.25)	4.	0.96^a^ (0.22)	6.	0.98^b^ (0.24)	7.
Hedonism	1.13^a^ (0.30)	3.	1.00^a,b^ (0.25)	6.	1.08 (0.24)	4.	1.07^b^ (0.23)	4.
Achievement	1.14^a,b,c^ (0.21)	2.	0.97^a^ (0.24)	7.	0.95^b^ (0.22)	7.	0.98^c^ (0.20)	6.
Power	0.90^a,b^ (0.27)	8.	0.85 (0.24)	9.	0.72^a^ (0.20)	9.	0.70^b^ (0.20)	10.
Security	0.99 (0.18)	7.	1.04 (0.16)	5.	1.01 (0.18)	5.	1.02 (0.14)	5.
Conformity	0.86^a^ (0.18)	9.	0.96^a,b^ (0.19)	8.	0.89 (0.20)	8.	0.89^b^ (0.18)	8.
Tradition	0.68^a^ (0.23)	10.	0.67^b^ (0.18)	10.	0.71 (0.19)	10.	0.80^a,b^ (0.19)	9.
Benevolence	1.08^a,b,c^ (0.18)	4.	1.15^a^ (0.14)	2.	1.21^b^ (0.14)	2.	1.17^c^ (0.13)	1.
Empathic guilt	4.12 (0.59)		4.12 (0.46)		4.26 (0.41)		4.12 (0.43)	
Norm-breaking guilt	2.61 (1.07)		3.09 (0.82)		2.68 (0.90)		3.40 (0.53)	
Shame	2.75 (0.70)		2.81 (0.65)		2.85 (0.72)		2.74 (0.49)	

*For each value means that share a superscript are significantly different at the p < 0.001 level.*

Across the samples, gender (1 = females; 2 = males) was positively related to achievement, power, and negatively to benevolence, universalism, empathic guilt, norm-breaking guilt, and shame (all *p*_s_ < 0.01). Age was positively related to universalism, security and conformity and negatively to hedonism, and achievement and norm-breaking guilt (all *p*_s_ < 0.01).

Table 4 indicates that the average proportion of reported behaviors in the four categories was fairly similar in the target countries, for secret transgressions in particular. A striking exception were interpersonal transgressions for which the percentage among Portuguese participants was clearly lower than in the other countries.

**TABLE 4:** Morally relevant behaviors in Bulgaria, Estonia, Finland, and Portugal, %.

Behavior/Country	Bulgaria	Estonia	Finland	Portugal
**Interpersonal transgressions**				
Hit sb. MB30	44	33	45	19
Dating two MB 29	30	25	60	14
Deceive partner MB17	68	36	54	22
Lie to get sex MB 14	23	10	11	2
Break promise MB 08	43	32	26	11
Interpersonal average %	41	27	39	14
**Prosocial behavior**				
Intervention MB28	52	68	14	27
Voluntary work MB12	59	58	42	55
Buy fair trade MB20	29	49	17	41
Move things MB04	76	55	51	58
Sort out biowaste MB15	95	48	29	58
Loan money	50	71	80	39
Prosocial average %	60	58	39	46
**Antisocial behavior**				
Break bottles MB02	29	14	11	18
Urinate publicly MB23	28	20	15	47
Cheat in exam MB03	72	83	51	78
Antisocial average %	43	39	26	48
**Secret transgressions**				
Read sms MB27	23	44	33	43
Poking around MB13	44	51	27	42
Talk behind back MB06	56	70	85	49
Secret average %	41	55	48	45

Table 5 shows the correlations of the four behavior categories with the higher order values and moral emotional categories in the whole sample. It indicates that somewhat surprisingly, self-transcendence–self-enhancement did not predict prosocial behaviors at all. Openness to change was positively related to all four morally relevant behavior categories, which suggests that people scoring high on openness tend to be more active in doing or at least reporting both good and bad actions. In line with this, conservation was negatively related to prosocial behaviors, which suggests that people scoring high on conservation tend to refrain from doing bad as well as good. Third, a look at the four behavior categories shows that the three transgression dimensions are similar in terms of their relations to self-enhancement, openness to change, and conservation, as well as to norm-breaking guilt. The more impersonal transgressions included in the antisocial dimension were negatively associated with both measures of guilt. Prosocial behaviors showed overall the weakest associations with value dimensions and measures of moral emotional tendencies. The finding that secret acts were the only behavior category that was related to tendency to report shame is consistent with shame being associated with public exposure (secret acts disclosed; Smith et al., 2002). Shame-prone individuals may be more inclined to remember their secret transgressions.

**TABLE 5:** The correlations of the four morally relevant behavior categories with higher-order values and moral emotional variables, the entire sample.

	Interpersonal	Prosocial	Antisocial	Secret
	transgressions	behaviors	behaviors	transgressions
Self-transcendence	−0.13**	0.01	−0.14**	−0.04
Self-enhancement	0.22**	0.00	0.16**	0.13**
Openness to change	0.17**	0.12**	0.12**	0.11**
Conservation	−0.18**	−0.11**	−0.09**	−0.11**
Empathic guilt	−0.06	0.01	−0.16**	−0.00
Norm-breaking guilt	−0.27**	−0.03	−0.16**	−0.10**
Shame	−0.00	−0.04	−0.08	0.17**

**p < 0.001.

The correlations of the number of self-reported MRB with values, guilt and shame in four countries, controlled for gender and age, are shown in Table 6. It indicates that hedonism showed the highest number of significant correlations with MRB across countries, followed by universalism, power, and conformity. All the significant correlations with hedonism, stimulation and power were positive, whereas conformity only showed significant negative correlations. Universalism also showed mostly negative significant correlations. To test the differences in correlations between samples we calculated *z*-tests, which showed that the correlation of stimulation with prosocial behavior was stronger in Estonia than in Finland (*z* = 2.022, *p* < 0.05) and with antisocial behavior stronger in Portugal than in Finland (*z* = 2.163, *p* < 0.05). Hedonism showed significantly higher correlation with interpersonal transgression in the Bulgarian sample than in the Finnish sample (*z* = 3.167, *p* < 0.01), and the correlation of power with interpersonal transgressions was higher in Bulgarian, Estonian and Portugal samples than in Finnish (*z* = 3.316, *p* < 0.01; *z* = 3.186, *p* < 0.01; *z* = 3.273, *p* < 0.01, respectively). For benevolence, the correlation with interpersonal transgressions was lowest in Bulgaria, differing from Finland (*z* = −2.806, *p* < 0.05). The norm-breaking guilt showed strongest negative correlation with interpersonal transgression in Bulgaria, which differed from zero correlation in Portugal (*z* = −2.785, *p* < 0.01). With secret transgressions, the norm-breaking guilt correlated lowest in Estonia compared to Finnish sample (*z* = −2.411, *p* < 0.05).

**TABLE 6:** The correlations of the number of self-reported moral behaviors with values, guilt, and shame in four countries controlling for gender and age.

		UN	SD	ST	HE	AC	PO	SE	CO	TR	BE	EG	NBG	Shame
Interpersonal transgressions	Bulgaria	−0.20*	0.06	0.22**	0.30**	0.09	0.26**	0.01	−0.30**	−0.10	−0.29**	−0.18*	−0.23**	0.01
	Estonia	−0.14*	0.07	0.12	0.19**	0.11	0.22**	−0.05	−0.21**	−0.10	−0.14*	−0.04	−0.18**	−0.00
	Finland	0.21*	0.00	0.17	−0.05	−0.03	−0.11	−0.16	−0.09	0.02	0.02	0.05	−0.11	−0.06
	Portugal	−0.16*	0.05	0.08	0.12	0.12	0.24**	−0.08	−0.11	−0.05	−0.15*	−0.10	0.06	0.06
Prosocial behavior	Bulgaria	0.11	−0.10	0.15	0.09	0.01	−0.05	0.00	−0.17*	−0.13	0.12	0.08	−0.02	−0.01
	Estonia	0.16	0.07	0.18**	−0.03	−0.12	−0.09	−0.01	−0.07	−0.07	0.05	−0.02	0.02	−0.07
	Finland	0.10	0.01	−0.03	0.08	−0.10	0.05	−0.07	−0.03	−0.14	0.11	0.08	0.06	0.01
	Portugal	0.08	0.10	0.14	0.07	−0.08	−0.00	−0.05	−0.14*	−0.06	0.08	0.15*	−0.00	0.01
Antisocial behavior	Bulgaria	−0.17*	0.08	0.18*	0.25**	0.06	0.19*	−0.08	−0.16	−0.17*	−0.02	−0.09	−0.26**	−0.06
	Estonia	−0.10	−0.10	0.10	0.19**	−0.03	0.00	−0.02	−0.05	0.05	0.08	−0.08	−0.18**	0.01
	Finland	0.02	−0.02	0.01	0.24**	−0.08	−0.01	0.01	−0.00	−0.09	−0.01	−0.09	−0.07	−0.02
	Portugal	−0.10	0.08	0.24**	0.31**	0.05	0.06	−0.12	−0.18*	−0.12	0.11	−0.06	−0.23**	0.05
Secret transgressions	Bulgaria	−0.06	0.03	0.13	0.21*	0.04	0.16	0.04	−0.21*	−0.08	−0.16*	−0.06	−0.16	0.17*
	Estonia	−0.11	−0.05	0.11	0.18**	0.09	0.13	−0.12	−0.12	−0.03	0.07	−0.05	−0.20**	0.17*
	Finland	−0.03	−0.06	0.07	0.21*	0.06	0.06	0.01	0.01	−0.17*	0.03	0.16	0.05	0.06
	Portugal	−0.08	−0.08	0.14	0.06	0.01	0.18*	0.03	−0.06	−0.02	−0.01	0.01	−0.05	0.26**

UN = universalism; SD = self-direction; ST = stimulation; HE = hedonism; AC = achievement; PO = power; SE = security; CO = conformity; TR = tradition; BE = benevolence; EG = Empathic guilt; NBG = norm-breaking guilt. *p < 0.05; **p < 0.01.

Interpersonal transgressions were predicted by the self-enhancement–self-transcendence contrast in three countries, except Finland, and by the openness to change–conservation contrast in Bulgaria and Estonia, where they were also associated with guilt over norm transgressions. Thus, Portugal was the only country to show approximative motivational continuity whereas a marked mixed pattern of correlations was evident in Bulgaria and Estonia. For prosocial behaviors no clear pattern emerged. The guilt and shame measures were not related to prosocial behaviors either, with the exception of empathic guilt in Portugal. Antisocial behavior was linked to the openness to change–conservation contrast in Bulgaria and Portugal, and showed an additional power–universalism contrast in Bulgaria. Hedonism was associated with antisocial behavior in Finland. Low norm-breaking guilt predicted antisocial behavior in three countries, not in Finland. Secret transgressions were predicted by power in Portugal and by hedonism in the three other countries. In Bulgaria we found both self-enhancement–self-transcendence and openness to change–conservation contrasts, in Finland only a weak openness–conservation contrast. An intriguing finding was the positive association of shame-proneness with secret transgressions in three countries.

In all, Table 6 indicates that the number (4) of motivationally heterogeneous patterns of value-behavior correlations equals the number (4) of bipolar, motivationally continuous patterns, which have been more or less the rule in earlier studies. Also three unipolar patterns (two for hedonism, one for power) were found. However, it should be noted that even in mixed patterns, motivational continuity is relatively high. For instance, for the interpersonal transgressions in Bulgaria and Estonia, the mixed pattern of correlations approximated the sinusoid curve (rhos = 0.87 and 0.84, *p* < 0.01), but not as well as did the homogeneous pattern in Portugal (rho = 0.98).

With regard to the strength of the associations of propelling and inhibiting values with behavior, the data in Table 6 are consistent with the previous findings by Schwartz et al. (2017) in that in only one of the 11 pairs of tradeoffs, the inhibiting value shows a higher correlation with the behavior category than did the promoting value, if we attend to the value showing the highest correlation and compare it with the correlation of the opposite value with the behavior. For instance, on the second row, interpersonal transgressions in Estonia, power, a propelling value, shows a correlation *r* = 0.22, and benevolence, the inhibiting value, *r* = −0.14. The mean difference in the magnitudes of 11 pairs of coefficients is 0.03. Moreover, all three unipolar patterns involve a propelling value.

We further examined the predictors of MRB by means of hierarchical regression analyses. Gender (male) and country (Bulgaria, Estonia, and Finland) were entered as dummy variables on the first step with age; values were entered on the second step and guilt and shame variables on the third step. To avoid the problem of multicollinearity, i.e., having opposite values that correlate highly negatively with each other simultaneously in a regression model, two models were calculated for every dependent variable. In the first model self-transcendence and conservation values were used and in the second model self-enhancement and openness-to-change values were entered because these value sets are located on different sides of the circumplex. Thus, altogether we ran eight regression analyses: two models for each of the dependent variables.

Summaries of the hierarchical regression analyses are reported in Table 7. Interpersonal transgressions were significantly predicted by age (positively), country (Bulgaria, Estonia, and Finland positively) and negatively by self-transcendence and conservation and positively by self-enhancement and openness-to-change values even when guilt and shame were taken into account. Norm-breaking guilt predicted the reported number of interpersonal transgressions negatively in both models. Altogether, the predictors explained 22% of the total variance of interpersonal transgressions.

**TABLE 7:** Summaries of the hierarchical regression analyses for self-reported moral behaviors.

	Interpersonal transgressions			Prosocial behaviors			Antisocial behaviors			Secret transgressions		
	Step1	Step2	Step 3	Step 1	Step 2	Step 3	Step 1	Step 2	Step 3	Step1	Step 2	Step 3
**Model 1**												
Gender (male)	0.09*	0.07*	0.07	0.01	0.02	0.03	0.26***	0.26***	0.23***	−0.12**	−0.13***	−0.11**
Age	0.04	0.09*	0.11**	0.10*	0.11**	0.10*	0.01	0.04	0.07	−0.15***	−0.12**	−0.08
Country (Bulgaria)	0.41***	0.35***	0.31***	0.20***	0.20***	0.21***	−0.11**	−0.15***	−0.20***	−0.01	−0.05	−0.10*
Country (Estonia)	0.20***	0.17***	0.14**	0.15**	0.14**	0.15**	−0.15**	−0.18***	−0.22***	0.26***	0.23***	0.18***
Country (Finland)	0.38***	0.38***	0.33***	−0.18***	−0.22***	−0.21***	−0.31***	−0.32***	−0.37***	0.10*	0.10*	0.05
Self-transcendence		−0.13***	−0.12**		0.11**	0.11**		−0.06	−0.04		−0.08*	−0.08*
Conservation		−0.15***	−0.12**		−0.11**	−0.12**		−0.11**	−0.07		−0.10**	−0.10**
Empathic guilt			−0.01			0.04			−0.02			−0.04
Norm-br. guilt			−0.12**			0.02			−0.17***			−0.10*
Shame			0.05			−0.03			0.03			0.20***
Adjusted R	0.18	0.21	0.22	0.11	0.14	0.14	0.13	0.14	0.17	0.06	0.07	0.11
Δ *R*^2^	0.18***	0.03***	0.01**	0.12***	0.02***	0.00	0.14***	0.01**	0.02***	0.06***	0.01**	0.04***
**Model 2**												
Gender (male)	0.09*	0.07*	0.07	0.01	0.02	0.03	0.26***	0.25***	0.23***	−0.12**	−0.14***	−0.11**
Age	0.04	0.10*	0.11**	0.10*	0.11*	0.10*	0.01	0.06	0.08	−0.16***	−0.11*	−0.07
Country (Bulgaria)	0.41***	0.34***	0.31***	0.20***	0.19***	0.20***	−0.11**	−0.16***	−0.20***	−0.01	−0.07	−0.11*
Country (Estonia)	0.20***	0.17***	0.14**	0.15**	0.14**	0.15**	−0.15**	−0.18***	−0.21***	0.25***	0.22***	0.18***
Country (Finland)	0.38***	0.35***	0.32***	−0.18***	−0.18***	−0.18***	−0.31***	−0.32***	−0.38***	0.10*	0.08	0.03
Self-enhancement		0.15***	0.14***		−0.05	−0.05		0.10**	0.08*		0.17***	0.16***
Openness to change		0.11***	0.09**		0.11**	0.12**		0.10**	0.07		0.06	0.06
Empathic guilt			−0.1			0.06			−0.02			−0.04
Norm-br. guilt			−0.10*			0.01			−0.15***			−0.08
Shame			0.05			−0.03			0.04			0.20***
Adjusted R	0.18	0.21	0.22	0.11	0.12	0.13	0.13	0.15	0.17	0.06	0.08	0.12
Δ *R*^2^	0.18***	0.04***	0.01*	0.12***	0.01**	0.00	0.14***	0.02***	0.02**	0.06***	0.03***	0.04***

*p < 0.05; **p < 0.01; ***p < 0.001.

For prosocial behaviors, moral emotions did not increase explained variance, but age (positively), country (Bulgaria and Estonia positively, Finland negatively) as well as values were significant predictors. Self-transcendence and openness to change values predicted positively and conservation negatively prosocial behavior. In the first model, the predictors explained 14% and in the second model 13% of the total variance.

Antisocial behaviors were strongly predicted by male gender, country (Bulgaria, Estonia, and Finland negatively) and norm-breaking guilt (negatively). Of the values, only self-enhancement was weakly related to it. The predictors explained 17% of the total variance in both models. Secret transgressions were significantly predicted by female gender, country (Estonia positively), and of the values self-transcendence and conservation negatively and self-enhancement positively. Norm-breaking guilt and shame were significant predictors in the first model and shame in the second model. Shame was the most powerful predictor in both models (both β_s_ = 0.20), and altogether the predictors explained 11 and 12% of total variance in the first and second model, respectively.

We also tested the potential moderating role of conformity in the value-behavior relations in each sample but did not find any effects.

## Discussion

The first research question concerned nature of the value-behavior relations. Four dimensions or categories of self-reported MRB were found by means of a factor analysis: interpersonal transgressions, prosocial behaviors, antisocial behaviors, and secret transgressions. While the value-behavior correlations for these four dimensions showed no strict uniformity across the four countries, a few generalizations are possible. Prosocial behaviors were linked to the self-transcendence–self-enhancement value dimension, except in Portugal where they had no associations with values. In contrast to the majority of previous findings, the three transgression categories, interpersonal and secret transgressions as well as antisocial behaviors, were equally likely to exhibit a mixed and a homogeneous pattern of correlations with values, i.e., both the self-transcendence–self-enhancement and the openness to change–conservation contrast simultaneously. This heterogeneous contrast was observed for all three transgression categories in Bulgaria and for the interpersonal one in Estonia. In Portugal and especially in Finland, the value–behavior correlations were lower and the patterns less clear-cut, although they were in line with the findings from the two other countries.

A fairly consistent finding was that values promoting behavior (self-transcendence values for prosocial behavior, openness to change values for the other three categories) were more strongly related to behavior than the values thwarting it. This was our second research question, and we largely corroborated the previous findings by Schwartz et al. (2017).

With regard to the moral emotional variables, our third research focus, the introduction of the guilt-over-norm-breaking measure to the TOSCA, to complement the empathy-based guilt measure, appeared useful, as this measure was more frequently (6 vs. 2 times out of 12) and more strongly (mean/r/’s = 0.15 vs. 0.08) associated with the three transgression categories than was empathic guilt. This finding highlights the important role that norms play in MRB. While empathic guilt did not add explanatory power beyond values in the hierarchical regression analyses, norm-breaking guilt did so in most of the analyses. Shame, on the other hand, was not associated with other types of MRB than secret transgressions, but it played an important role in explaining these transgressions.

That secret transgressions formed a category of their own was an unexpected finding. While secret transgressions were associated with hedonism in Finland, in the three other countries they were positively related to power. In Bulgaria and Estonia, a mixed pattern of correlations similar to the other two morally negative categories was found.

What do the present exploratory findings tell us generally about moral behavior? The relatively high proportion of motivationally mixed correlation patterns in our data was striking, against the background of previous research focused on finding motivationally pure behavior. Instead of being simply an issue of egoism vs. altruism or breaking norms vs. following them (hedonism–conformity), moral behavior often seems to involve both those aspects, if our findings lend themselves to be replicated.

What do our findings tell us about moral values? A look at values in terms of the number of their correlations with the behavioral categories (Table 6) indicates that self-direction, achievement and security had no correlations. Among conservation values, conformity had the highest number correlations, 6, which is consistent with the idea that control of norm-breaking is one central function of morality (Helkama, 2011; Vauclair et al., 2014). Thus, the present study suggests that conformity is a more prototypical moral value than are the other conservation values, and hedonism and stimulation are more apt to motivate (or justify) norm-breaking than the other openness-to-change value, self-direction.

We replicated the Schwartz et al. (2017) finding that propelling values tended to be more strongly associated with behavior than were inhibiting values, and unlike theirs, our design perhaps allowed both types of values a fair chance. According to our results, supporters of conservation values reported not only fewer transgressions but also fewer prosocial acts. That conservation values (conformity in particular) might be inherently inhibiting is suggested by the findings from a study by Leikas et al. (2009). They investigated the relations of values and regulatory focus (Higgins, 1998) and found that conformity was related to prevention focus and a prevention-framed persuasive message evoked more compliance among adherents of conformity and security values. However, Bilsky et al. (2020) found, in their study of relations of values to attitudes toward norms, that (positive) correlations for conformity were systematically higher than (negative) correlations for the opposite value, stimulation. This finding speaks against the idea that conformity would be an inhibitory value by nature. For norm-following, conformity seems to be a propelling/promoting value.

Secret transgressions were consistently correlated with power, which suggests that domination of others is associated with admitting that one has penetrated into their privacy. An intriguing finding was the association of shame-proneness with secret transgressions. Research suggests that people who value power are not likely to feel shame (Helkama et al., 2018), so this combination seems to be counterintuitive to some extent. Shame is linked to a desire to hide and has been found to be associated with poor self-regulation (Woien et al., 2003) and also with public exposure (Smith et al., 2002). We may speculate that this kind of concern with secrecy in the sense of hiding and being publicly exposed gives rise to the salience of the schema of secret acts (because prying into the (secret) affairs of others is also done in secret). This concern with secrets, combined with poor self-regulation, may thus be salient for shame-prone individuals and explain the relation of shame and admitting breaches of secrecy. The issue deserves further study, in which a more differentiated measure of shame proneness, such as the GASP (Cohen et al., 2011) could be used. Is the ambivalent combination of power and shame limited to partner relationships characterized by jealousy, or could it be behind breaches of privacy more generally?

In the social psychology and sociology of morality, interest in secrets has been next to non-existent, as one looks in vain for the term in the handbooks. In fact, the only reference to it we found in psychological literature on moral judgments was 50 years old. Von Wright and Niemelä (1966) asked individuals from different age groups to assess a number of acts (e.g., puncturing a tire of another child’s bike but regretting afterward) in terms of in terms of their similarity. Among the 7-year-olds, one of the three basic dimensions was doing something in secret, keeping something secret (the others were physical violence and stealing, whereas for adults, the dimensions were totally different (irresponsible behaviors, behaviors that produced gain to the perpetrator, and deceiving other people); von Wright, 1970). This gives us reason to believe that our finding on secret acts is not just a fortuitous one but reflects something more fundamental in human morality.

Indeed, keeping (and disclosing) secrets is a pervasive phenomenon in social life (Slepian et al., 2017; Slepian and Greenaway, 2018) from the deepest corners of a person’s intimate self to professional, e.g., doctor-patient, relationships and trade secrets. Anton Chekhov (1899/1986, p. 577) makes Gurov, the protagonist of his famous story *Lady with lapdog*, muse: “The whole private personal life is kept a secret, and perhaps that is partly the reason why civilized individuals are so anxious that their personal secrets should be respected.” Writing about medical secrecy, Raymond Villey (1986, p. 163) states that keeping secrets is not only a necessary condition of trust but also a symbol of the respect that the physician owes to her or his patient. Schwartz added *privacy* to the 1995 version of his value survey. While this item turned out to be neither motivationally pure nor having culturally invariant meaning, in the Finnish national value measurements its importance has been fairly high (16-19/57), higher than wisdom, for instance (Puohiniemi, 2006). In 1999, privacy was a predominantly conservation value in Finland, but by 2015, its meaning had shifted toward self-direction (Puohiniemi and Helkama, 2018). Thus, not respecting other persons’ privacy is tantamount to acting against an important value.

The fourth question addressed in this study were cross-cultural differences. The value priorities in three of the four target countries, Estonia, Finland, and Portugal, were close to the pan-cultural consensus (Schwartz and Bardi, 2001), with benevolence, self-direction, and universalism on the top and tradition and power on the bottom. The Bulgarian sample differed from the others in its emphasis on achievement and hedonism. In view of the current theorizing about cross-cultural differences in value-behavior linkages, our findings are somewhat paradoxical. If we assume that those linkages are stronger in individualistic societies than in collectivistic ones, then the strongest value-behavior associations should be found in the most individualistic country, Finland, and the weakest ones in the most collectivistic Bulgaria. The examination of the cultural tightness-looseness scores (Uz, 2015) of the four countries leads to the expectation that the associations would be strongest in Portugal, the loosest country, and weakest in Estonia, the tightest country. In fact, our results go against both expectations, because the strongest and most consistent links were observed in Bulgaria and Estonia, and the weakest ones in Finland and Portugal. As was the case in the Schwartz et al. (2017) study in Italy, Poland, Russia, and the United States, the country scores on the Gelfand et al. (2011) and the Uz (2015) were in conflict in our study, too, and were not helpful in accounting for differences in the strength of value-behavior relations.

However, a comparison of the value hierarchies of the samples shows that the Bulgarian sample was the most individualistic one, with self-direction and achievement as its top values, and also loosest in terms of value dispersion. This could explain the strong associations. But the associations were almost as strong in Estonia, which on the sample level was quite similar to Finland and Portugal in terms of value hierarchy and close to Finland on tightness-looseness. To understand why behavior links were stronger in Estonia and Bulgaria than in Finland and Portugal, we could speculatively appeal to the communist past of those two countries. In ex-communist countries, individualism may to a great extent be associated with breaking social norms while in older democracies it would be easier to combine individualistic values with following norms. Gelfand et al.’s (2011) categorization of Estonia as a particularly loose culture was based on Estonians’ perception of looseness of their country, which is not in line with the characteristics of our Estonian sample. Anyway, these findings suggest that while it is important to take the tightness-looseness in the specific samples into account, the overall social representation of the national culture on this dimension plays a role, too.

The fact that the respondents were (primarily female) university students is naturally a serious limitation of the present study. In further studies, more gender-balanced samples should be used. Also the fact that of the 30 items in the original MRB scale as many as 13 were discarded in the process of analysis suggests that our attempt to sample actions that would be representative of the morally relevant actions in the life of students was not entirely successful. Moreover, it would have been preferable to arrange a four-country brainstorming session to construct the MRB questionnaire instead of using the version that was produced in Finland. However, as this study was exploratory and aimed at producing new ideas, from an inductive starting point, the findings that were consistent across most of the four countries would suggest that they are worth pursuing further, with more diverse and representative samples and new questionnaires. One promising direction is the examination of the significance of secrets in the social psychology of morality. The right to have secrets is one of the bases of freedom, democracy and rule of law and deserves more attention. Is the finding that shame-prone individuals who have high regard for power are more likely than others to report breaches of privacy replicable in other contexts? As power and the proclivity to feel shame have been found to correlate negatively, it would be interesting to delve deeper into this issue. Another issue that seems to deserve further scrutiny is the finding that our inductively derived morally relevant behavior categories were largely motivationally mixed in relation to the Schwartz value model, in contrast to majority of previous findings on value-behavior associations.

## Data Availability Statement

The raw data supporting the conclusions of this article will be made available by the authors, without undue reservation.

## Ethics Statement

The patients/participants provided their oral informed consent to participate in this study.

## Author Contributions

LM, KH, and MS-K: conceptualization, study design, and funding acquisition. LM, MS-K, JV, KP, and KL: data collection and analysis and providing critical revision. LM and KH: writing – original draft. All authors contributed to the article and approved the submitted version.

## Conflict of Interest

The authors declare that the research was conducted in the absence of any commercial or financial relationships that could be construed as a potential conflict of interest.

## Footnotes

1 The data from Bulgaria, Finland and Portugal were used by Helkama et al. (2018), without the MRB questionnaire, and the analysis focused on relations of values to moral emotions.
